# Effects of adipocyte-conditioned cell culture media on S1P treatment of human triple-negative breast cancer cells

**DOI:** 10.1371/journal.pone.0286111

**Published:** 2023-05-23

**Authors:** Xiyuan Wu, Martin Wabitsch, Jian Yang, Meena Kishore Sakharkar

**Affiliations:** 1 College of Pharmacy and Nutrition, University of Saskatchewan, Saskatoon, SK, Canada; 2 Department of Pediatrics and Adolescent Medicine, Ulm University Medical Center, Ulm, Germany; Royal Adelaide Hospital, AUSTRALIA

## Abstract

Sphingosine-1-phosphate (S1P) is a potent sphingolipid metabolite that regulates a wide range of biological functions such as cell proliferation, cell apoptosis and angiogenesis. Its cellular level is elevated in breast cancer, which, in turn, would promote cancer cell proliferation, survival, growth and metastasis. However, the cellular concentration of S1P is normally in the low nanomolar range, and our previous studies showed that S1P selectively induced apoptosis of breast cancer cells at high concentrations (high nanomolar to low micromolar). Thus, local administration of high-concentration S1P alone or in combination of chemotherapy agents could be used to treat breast cancer. The breast mainly consists of mammary gland and connective tissue stroma (adipose), which are dynamically interacting each other. Thus, in the current study, we evaluated how normal adipocyte-conditioned cell culture media (AD-CM) and cancer-associated adipocyte-conditioned cell culture media (CAA-CM) would affect high-concentration S1P treatment of triple-negative breast cancer (TNBC) cells. Both AD-CM and CAA-CM may suppress the anti-proliferative effect and reduce nuclear alteration/apoptosis caused by high-concentration S1P. This implicates that adipose tissue is likely to be detrimental to local high-concentration S1P treatment of TNBC. Because the interstitial concentration of S1P is about 10 times higher than its cellular level, we undertook a secretome analysis to understand how S1P would affect the secreted protein profile of differentiated SGBS adipocytes. At 100 nM S1P treatment, we identified 36 upregulated and 21 downregulated secretome genes. Most of these genes are involved in multiple biological processes. Further studies are warranted to identify the most important secretome targets of S1P in adipocytes and illustrate the mechanism on how these target proteins affect S1P treatment of TNBC.

## Introduction

Sphingosine-1-phosphate (S1P) is the last metabolite in the highly conserved sphingolipid metabolism pathway. Together with ceramide, it forms a rheostat to regulate a wide range of biological, physiological and pathophysiological processes [1–3]. S1P is synthesized by phosphorylation of sphingosine, which is catalyzed by sphingosine kinase (SphK). Two types of SphK have been discovered so far, with SphK1 existing in the cytosol and SphK2 existing in the nucleus or perinuclear region [4, 5]. S1P synthesized by SphK1 is transported out of the cells to interact with a family of cell-surface G protein-coupled receptors (S1PR_1-5_). This “inside-out signaling” activates and/or promotes vast biological functions such as cell proliferation and survival, angiogenesis and T-cell migration [6–9]. Comparing to SphK1, the function of SphK2 is relatively less known. However, there are evidences showing that SphK2 can induce cell apoptosis via different mechanisms, including inhibiting histone deacetylase (HDAC) 1 and 2 and interacting with B-cell lymphoma extra large (Bcl-xL) [10–12].

SphK1 is overexpressed in breast cancer cells. Elevated S1P will interact with S1PR_1_ and S1PR_3_ and activate PI3K/Akt, Ras/ERK, PLC/PKC and Rho/ROCK signaling pathways [13–17], which, in turn, would promote proliferation, survival, growth, migration and metastasis of breast cancer cells [13, 18, 19]. SphK1/S1P has also been observed to interact with other signaling pathways such as estrogen receptor (ER) and epidermal growth factor receptor (EGFR) [17, 20–23]. The SphK1-S1P-S1PR_1_ axis has emerged to be one of the major targets in developing anticancer agents against breast cancer, and quite a few SphK1 inhibitors such as SK1-I and S1PR_1_ antagonists such as NIBR0213 have been developed [24, 25]. The cellular concentration of S1P is in the low nanomolar range, which is much lower than its serum level (~0.5 μM) [26, 27]. Thus, the downregulation of S1P-S1PR_1_ signaling by SphK1 inhibitors or S1PR_1_ antagonists is limited in a very narrow range with low S1P concentration. However, our previous studies showed that high concentration of S1P (high nanomolar to low micromolar) can selectively kill breast cancer cells [28–30]; however, the underlying mechanism is unknown. Our preliminary study on the mechanism showed that S1P could enter into breast cancer cells and accumulate in the perinuclear region (data unpublished). Regardless, our previous studies imply that local administration of excess S1P may not only inhibit breast cancer growth but also be associated with low adverse drug reactions (ADRs) as residual S1P could be absorbed into the bloodstream.

**Fig 1:**
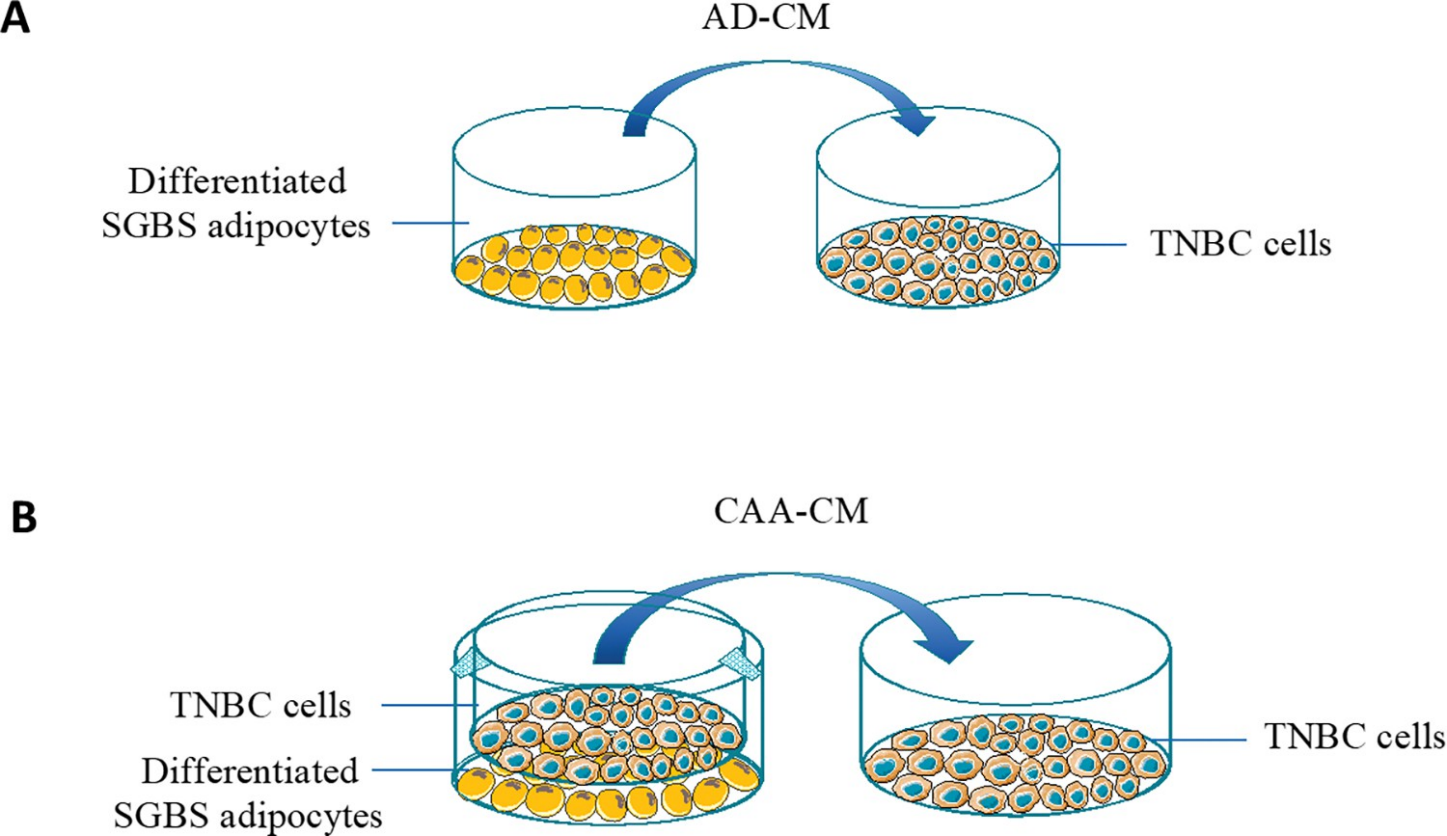
Schematic representation of a transwell co-culture protocol used to study the effects of (A): normal adipocyte-conditioned cell culture media (AD-CM) and (B): cancer-associated adipocyte-conditioned cell culture media (CAA-CM) on high-concentration S1P treatment of human triple-negative breast cancer (TNBC) cells.

As a specialized female exocrine organ, the breast is mainly divided into ductal epithelium, which forms the mammary gland, and connective tissue stroma, which is composed predominantly of adipose tissue and dynamically interacts with the ductal epithelium [31, 32]. It has been shown that obesity can induce breast cancer via activation of different oncogenic pathways such as PI3K/Akt pathway [33–35] and adipocytes can increase breast cancer cell survival and reduce chemotherapy efficacy [36–38]. Furthermore, S1P concentration in the breast interstitial fluid is about 10 times of its cellular concentration [39], suggesting that S1P may play an important role in modelling the extracellular matrix (ECM) and regulate the communications between mammary gland and stromal adipose tissue. Thus, in the current study, we undertook a co-culture protocol (Fig 1) to investigate whether adipocyte-conditioned cell culture media would affect high-concentration S1P treatment of triple-negative breast cancer (TNBC) cells, the most-sensitive subtype of human breast cancer cells.

## Results and discussion

### Effects of adipocyte-conditioned cell culture media on S1P treatment of breast cancer cells

**Fig 2:**
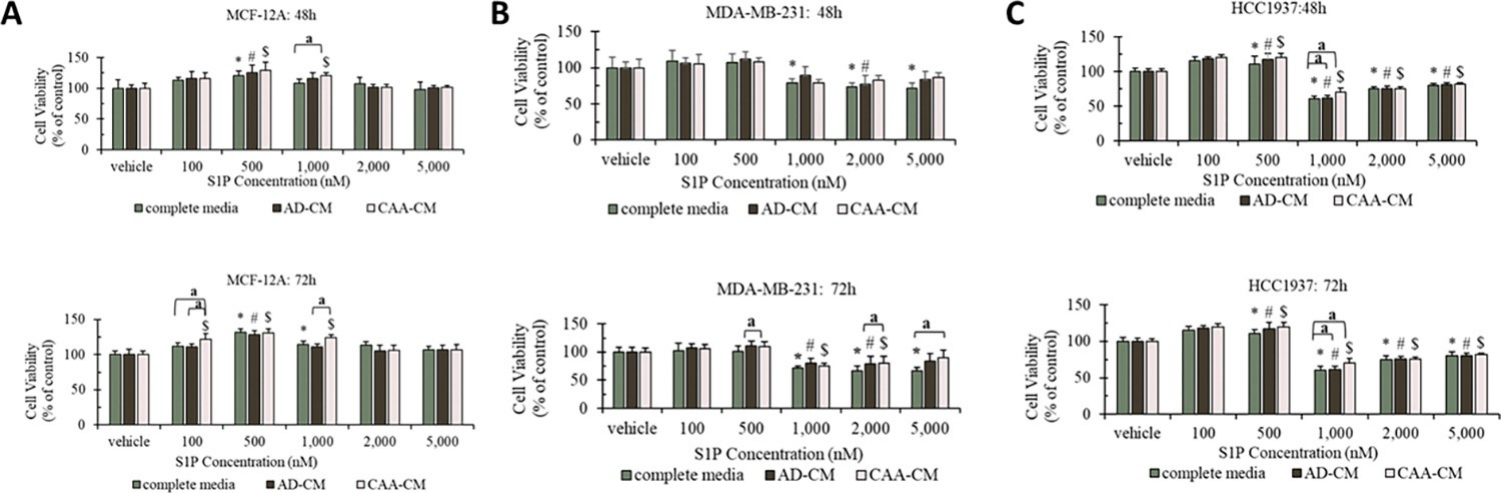
Cell viability (% of vehicle control) of normal mammary gland epithelial cell line MCF-12A (A) and triple-negative breast cancer (TNBC) cell lines MDA-MB-231 (B) and HCC1937 (C) under S1P treatment (concentration: 100–5000 nM) in complete media (control), AD-CM and CAA-CM for 48 h and 72 h, respectively. Cell treatment with methanol was used as the vehicle control. The cell viability was measured in triplicate (n = 3) using the MTT assay. The experimental data were analyzed by one-way ANOVA for each individual type of cell culture media series and two-way ANOVA followed by Tukey’s post-hoc test on three types of cell culture media series. A significant difference was defined by *p* < 0.05 compared to the vehicle control under different types of cell culture media series (*complete media, ^#^AD-CM and ^$^CAA-CM). Under the same S1P concentration, a represents a significant difference (*p* < 0.05) between the indicated two groups.

As mentioned above, the mammary gland constantly and dynamically interacts with the surrounding stromal adipose tissue, and adipocytes in the tumor ECM can not only promote proliferation and survival of breast cancer cells but also reduce chemotherapy efficacy [32–38]. It is rational to believe that adipocytes would also affect the anti-proliferative and apoptotic effects of high-concentration S1P against human breast cancer cells. Thus, we decided to evaluate the effects of both normal adipocyte-conditioned cell culture media (AD-CM) and cancer-associated adipocyte-conditioned cell culture media (CAA-CM) on S1P treatment of two TNBC cell lines MDA-MB-231 and HCC1937 using normal mammary gland epithelial cell line MCF-12A as a control (Fig 2).

As shown in Fig 2A, towards MCF-12A cells, S1P imposed a bell-shaped response on proliferation of the MCF-12A cells with the maximum effect achieved at 500 nM. It is noticed that prolonged treatment would further increase the proliferative effect of S1P. Cell viability of MCF-12A cells was increased by 32% after treatment with 500 nM S1P for 72 h. AD-CM did not alter cell viability of the MCF-12A cells towards S1P treatment at any tested concentration (*p* > 0.05). CAA-CM could enhance the proliferative effect of S1P at concentrations between 100 and 1000 nM. Cell viability of the MCF-12A cells was significantly increased (*p* < 0.05) at 1000 nM S1P for both 48 h and 72 h treatments and 100 nM S1P for 72 h treatment. However, the change of cell viability was less than 10% for these treatments. Thus, we may conclude that neither AD-CM nor CAA-CM can impose a dramatic change on the effects of S1P against the MCF-12A cells.

For the MDA-MB-231 cells, its cell viability was significantly decreased (*p* < 0.05) by S1P at concentrations higher than 1000 nM and prolonged treatment enhanced the effect (Fig 2B). AD-CM slightly reduced the anti-proliferative effect of S1P; however, the decrease was not statistically significant. CAA-CM could also alleviate the anti-proliferative effect of S1P; however, the decrease was only significant (*p* < 0.05) at treatment of 5000 nM for 72 h. As for the HCC1937 cells, we observed similar phenomenon that S1P suppressed cell proliferation at concentrations higher than 1000 nM (Fig 2C). Prolonged treatment did not intensify the effect. Maximum inhibition of HCC1937 cell proliferation was achieved at 1000 nM with cell viability reduced by more than 39%. Neither AD-CM nor CAA-CM elicited any effect on the anti-proliferative function of S1P except a slight decrease (< 10%) at S1P concentration of 1000 nM (*p* < 0.05).

Based on the above analyses, we conclude that S1P can increase cell proliferation of MCF-12A cells at concentrations between 500 and 1000 nM but decrease cell proliferation of both MDA-MB-231 and HCC1937 cells at concentrations higher than 1000 nM. Both AD-CM and CAA-CM might suppress the anti-proliferative function of S1P towards MDA-MB-231 and HCC1937 cells, however, these effects are relatively mild (only at 5000 nM S1P and 72 h treatment for MDA-MB-231 cells and 1000 nM S1P and 48h and 72 h treatments for HCC1937). Further mouse xenograft study is warranted to confirm whether adipocytes could impose a significant impact on the anti-proliferative effect of S1P against breast cancer cells at high concentrations (high nM to low μM).

### Effects of adipocyte-conditioned cell culture media on nucleic alteration caused by S1P

Our previous studies have shown that S1P can selectively induce apoptosis of breast cancer cells at high concentrations (high nanomolar to low micromolar range) [28–30]; and our preliminary Raman spectroscopic studies showed that S1P tends to accumulate inside the cancer cells, especially around the perinuclar region (unpublished data). Therefore, the apoptotic effect associated with high-concentration S1P is likely through its intracellular functions and independent of the cell-surface S1PRs. In this study, we used DAPI staining method to evaluate how AD-CM and CAA-CM would affect the apoptotic effect of high-concentration S1P.

**Fig 3:**
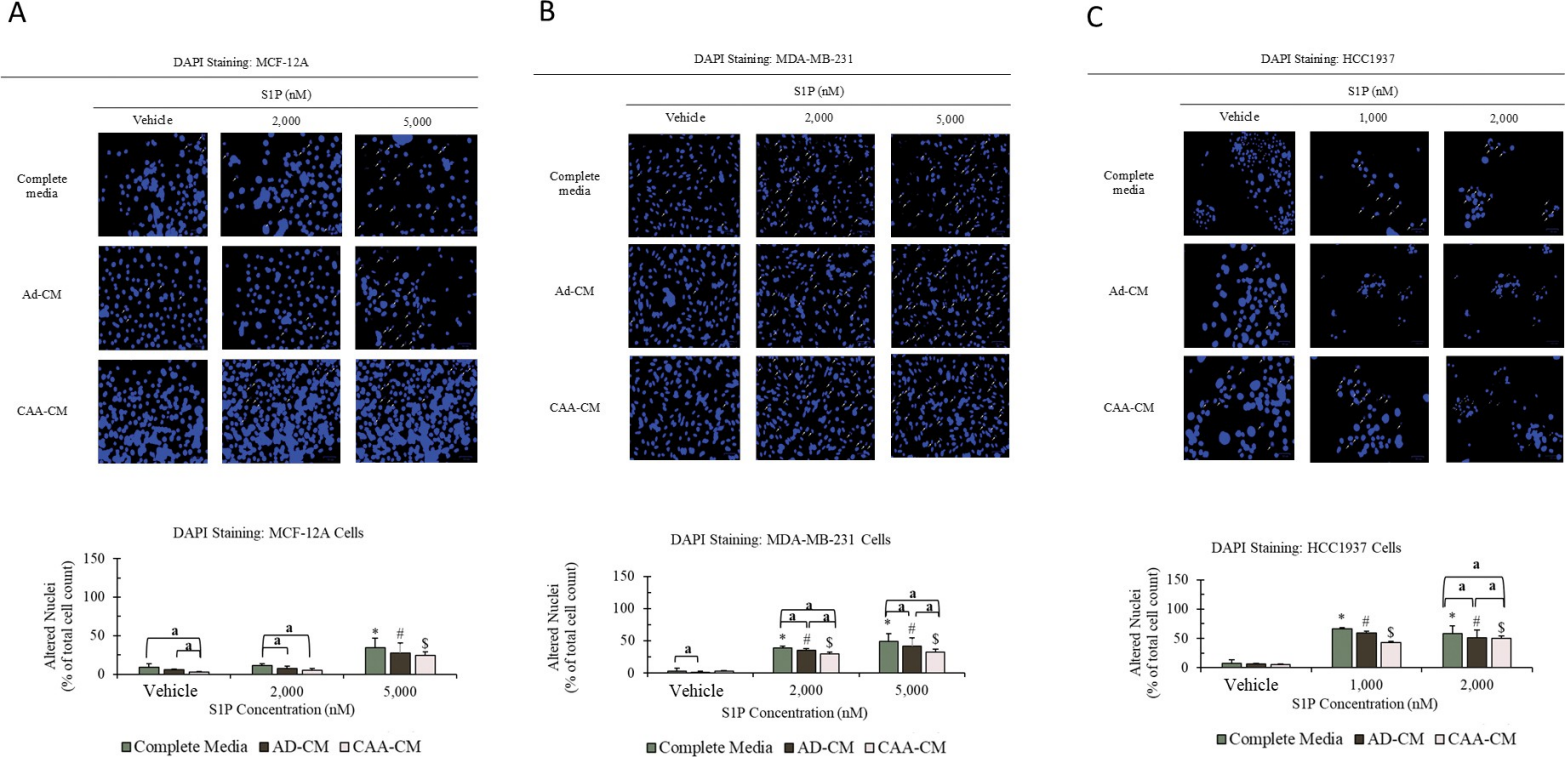
Morphological change of nucleus (DAPI staining: shown in small white arrows) and percentage of nucleic alteration in MCF-12A (A), MDA-MB-231 (B) and HCC1937 (C) cells under different treatment conditions for 48 h. The MCF-12A and MDA-MB-231 cells were treated with 2000 and 5000 nM S1P, respectively, in complete media, AD-CM or CAA-CM; whereas the HCC1937 cells were treated with 1000 and 2000 nM S1P, respectively, in complete media, AD-CM or CAA-CM. This experiment was carried out in triplicate and cells treated with methanol were used as the vehicle control. Percentage of nucleic alteration (mean ± SD) was calculated by dividing the number of cells with chromatin condensation, nuclear fragmentation and nuclear condensation over the total number of cells. The experimental data were analyzed by one-way ANOVA for each individual type of cell culture media series and two-way ANOVA followed by Tukey’s post-hoc test on three types of cell culture media series. A significant difference was defined by *p* < 0.05 compared to the vehicle control under different types of cell culture media series (*complete media, ^#^AD-CM and ^$^CAA-CM). Under the same S1P concentration, “a” represents a significant difference (*p* < 0.05) between the indicated two groups.

As shown in Fig 3, S1P caused morphological alteration of nuclei in both normal mammary gland epithelial MCF-12A cells and breast cancer MDA-MB-231 and HCC1937 cells; however, the effect was much stronger towards the cancer cells. Furthermore, both AD-CM and CAA-CM were capable of reducing the nucleic alteration caused by S1P. For the MCF-12A cells (Fig 3A), significant nucleic alteration of 34.3% was only observed at S1P concentration of 5000 nM; and AD-CM and CAA-CM decreased the nucleic alteration down to 27.8% and 24.7%, respectively. However, these reductions were not statistically significant. For the MDA-MB-231 cells (Fig 3B), significant nucleic alterations were observed at both S1P concentrations. At 2000 nM S1P, the nucleic alternation was at 39.3% in complete media. Addition of AD-CM and CAA-CM brought nucleic alteration down to 34.8% (*p* < 0.05) and 29.8% (*p* < 0.05), respectively. At 5000 nM S1P, the nucleic alteration was 48.8% in complete media, while the addition of AD-CM and CAA-CM reduced it to 41.3% (*p* < 0.05) and 32.8% (*p* < 0.05), respectively. For the HCC1937 cells (Fig 3C), significant nucleic alteration was also observed at both S1P concentrations. At 1000 nM S1P, nucleic alteration was decreased from 66.3% in complete media to 59.6% in AD-CM and 42.7% in CAA-CM, respectively. However, these reductions in nucleic alteration were not statistically significant. At 2000 nM S1P, nucleic alteration was statistically significantly decreased from 58.6% in complete media to 50.9% in AD-CM (*p* < 0.05) and 50.2% in CAA-CM (*p* < 0.05), respectively.

The above analyses suggest that S1P can selectively induce apoptosis in TNBC cells at high concentrations (> 1000 nM) and this effect is likely via its intracellular functions. Adipocyte-conditioned cell culture media AD-CM and CAA-CM can reduce nucleic alteration/apoptosis caused by high concentrations of S1P in TNBC cells, and CAA-CM is more potent than AD-CM. Thus, we may conclude that the presence of adipose tissue is detrimental to local high-concentration S1P treatment of human triple-negative breast cancer.

### Secretome analysis of differentiated SGBS adipocytes treated with S1P

**Fig 4:**
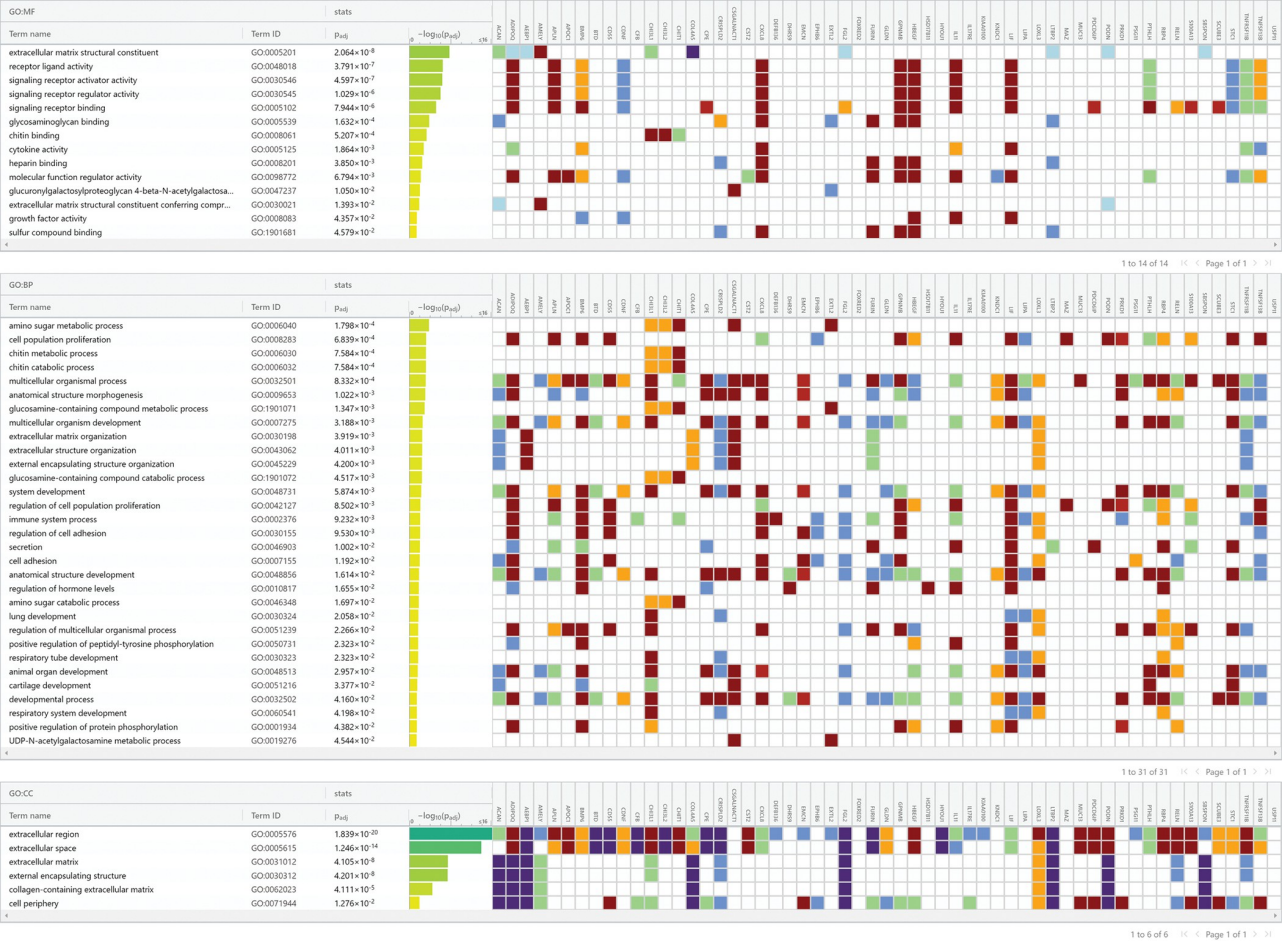
Functional profiling (MF: molecular function; BP: biological process; and CC: cellular component) of the 57 secretome DEGs in differentiated SGBS adipocytes treated with 100 nM S1P. This figure was generated using g:Profiler (https://biit.cs.ut.ee/gprofiler/gost). The color scheme used for the figure can also be found at the g:Profiler website.

The above adipocyte-conditioned cell culture media were prepared in absence of S1P treatment. Since interstitial S1P concentration is almost 10 times of its cellular concentration, it is critical to investigate how S1P would affect the profile of secreted protein molecules for the adipocytes. Thus, we undertook a transcriptome study of the differentiated SGBS adipocytes treated with 100 nM S1P [40]. Using |Fold Change| ≥ 1.50 and *p* < 0.05, we identified a set of differentially expressed genes (DEGs), which were subsequently matched to human secretome gene list [41]. As shown in Table 1, 36 secretome genes were upregulated and 21 secretome genes were downregulated, respectively, in responding to S1P treatment. These genes play important roles in not only normal biological and physiological functions but also carcinogenesis and cancer progression. For example, overexpression of *MUC13*, encoding mucin 13, enhances carcinogenesis and cancer progression [42, 43]. However, the regulatory functions of the most upregulated gene *CST2* and most downregulated gene *HYOU1* are less known in breast cancer. Gene *CST2* was observed to be highly overexpressed in breast cancer tissues and could be used as a marker for breast cancer diagnosis [44]. Gene *HYOU1*, which possesses anti-apoptotic functions, was also upregulated in breast cancer [45, 46]. Therefore, we did a functional profiling analysis of the 57 secretome DEGs using g:Profiler (https://biit.cs.ut.ee/gprofiler/gost). As shown in Fig 4, almost all of genes are involved in multiple biological processes. This suggests that S1P may regulate multiple signaling pathways in the differentiated SGBS adipocytes and further studies are warranted to identify the most important targets of S1P in adipocytes and figure out the mechanism on how S1P regulate the dynamic bidirectional communication between adipocytes and mammary gland epithelial cells.

**Table 1:** Upregulated and downregulated genes encoding secreted proteins in differentiated SGBS adipocytes treated with 100 nM S1P (n = 3).

**Upregulated Genes**			
Gene name	Fold change	Gene name	Fold change
*CST2*	2.06	*LIPA*	1.58
*AMELY*	1.98	*APLN*	1.57
*CXCL8*	1.93	*TNFSF13B*	1.57
*STC1*	1.88	*BTD*	1.56
*MUC13*	1.82	*SBSPON*	1.56
*IL11*	1.78	*CSGALNACT1*	1.55
*BMP6*	1.75	*DHRS9*	1.55
*CHI3L2*	1.75	*LOXL3*	1.54
*LIF*	1.75	*CHIT1*	1.53
*EMCN*	1.74	*EXTL2*	1.53
*ADIPOQ*	1.70	*HBEGF*	1.52
*CPE*	1.65	*PTHLH*	1.52
*CHI3L1*	1.61	*RBP4*	1.52
*HSD17B11*	1.61	*USP11*	1.52
*FGL2*	1.60	*CRISPLD2*	1.51
*GPNMB*	1.60	*KNDC1*	1.51
*APOC1*	1.59	*CD55*	1.50
*DEFB136*	1.59	*PDCD6IP*	1.50
	**Downregulated Genes**		
Gene name	Fold change	Gene name	Fold change
*HYOU1*	-2.14	*CFB*	-1.69
*SCUBE3*	-1.97	*FURIN*	-1.69
*GLDN*	-1.95	*KIAA0100*	-1.69
*PODN*	-1.89	*PRKD1*	-1.65
*ACAN*	-1.87	*FOXRED2*	-1.63
*LTBP2*	-1.83	*EPHB6*	-1.60
*COL4A5*	-1.79	*S100A13*	-1.60
*TNFRSF11B*	-1.78	*AEBP1*	-1.54
*IL17RE*	-1.76	*MAZ*	-1.53
*RELN*	-1.72	*PSG11*	-1.51
*CDNF*	-1.70		

## Materials and methods

### Materials

Almost all chemicals used in the current study, including sphingosine-1-phosphate (S1P) and 3-(4,5-dimethylthiazol-2-yl)-2,5-diphenyltetrazolium bromide (MTT), were purchased from Sigma-Aldrich Canada (Oakville, ON, Canada). DAPI (4’,6-diamidino-2-phenylindole dihydrochloride) was purchased from Thermo Fisher Scientific (Burlington, ON, Canada). Human mammary gland epithelial cell line MCF-12A and triple-negative breast cancer cell lines MDA-MB-231 and HCC1937 were purchased from the American Type Culture Collection (ATCC, Manassas, VA, USA). Human preadipocyte cell line SGBS was established in co-author Martin Wabitsch’s laboratory. Dulbecco’s Modified Eagle’s Medium (DMEM), 1:1 mixture of DMEM with Ham’s F12 medium (DMEM-F12), Leibovitz’s L-15 Medium and RPMI-1640 Medium were purchased from Gibco (Thermo Fisher Scientific, Canada).

### Generation of adipocyte-conditioned cell culture media

Differentiation of SGBS pre-adipocytes was reported in our previous study [40]. When SGBS pre-adipocytes were differentiated to day 14, a 6-well transwell co-culture system (Fig 1) was used for generating normal adipocyte-conditioned cell culture media (AD-CM) and cancer-associated adipocyte-conditioned cell culture media (CAA-CM). For CAA-CM, either MDA-MB-231 or HCC1937 cells were plated onto the upper chamber of the transwell insert (pore size: 0.4 μm) at a density of 1 × 10^5^ cells/well and transferred to plate wells with differentiated SGBS adipocytes in the bottom. This co-culture system was cultivated using DMEM containing 1% FBS for 3 d. Subsequently, the cell culture media were collected by centrifuging at 10000 g for 15 min, filter-sterilized (pore size: 0.2 μm), and stored at -80°C for further experimental analyses.

### Cell viability assay

MTT assay was used to evaluate the effects of adipocyte-conditioned cell culture media on S1P treatment of TNBC cell lines MDA-MB-231 and HCC1937. Normal mammary gland epithelial cell line MCF-12A was used as a control. For each cell line, the cells were cultured in 96-well plates with normal complete growth media, AD-CM with 10% FBS and CAA-CM with 10% FBS, respectively. The respective seeding number was 5 x 10^3^ cells/well for MCF-12A, 5 x 10^3^ cells/well for MDA-MB-231, and 1 x 10^4^ cells/well for HCC1937. The cells were cultured for 24 h for attachment before being treated with S1P (final concentrations: 100, 500, 1000, 2000 and 5000 nM) for 48 h and 72 h, respectively. Since methanol was used as the solvent for S1P, cells treated with methanol (final concentration: 1%) were used as a vehicle control. The detailed MTT assay protocol can be found in our previous study [40].

### Nuclear alteration analysis

DAPI staining was used to analyze the effects of adipocyte-conditioned cell culture media on S1P-induced nuclear alterations in MCF-12A, MDA-MB-231 and HCC1937 cells. For each cell line, cells were cultured in 24-well plates with normal complete growth media, AD-CM with 10% FBS and CAA-CM with 10% FBS, respectively. The respective seeding umber was 1 x 10^5^ cells/well for MCF-12A, 1.5 x 10^5^ cells/well for MDA-MB-231 and 2 x 10^5^ cells/well for HCC1937. The cells were cultured for 24 h for attachment before being treated with S1P for 48 h before staining. S1P concentration was 2000 and 5000 nM for MCF-12A, 2000 and 5000 nM for MDA-MB-231, and 1000 and 2000 nM for HCC1937, respectively. Cells treated with methanol (used to dissolve S1P) were used as a vehicle control. After incubation, the cells were first fixed in 4% paraformaldehyde for 20 min and rinsed with 1X PBS twice. Then, the cells were permeabilized in 0.1% Triton X-100 for 15 min and rinsed with PBS twice. After permeabilization, DAPI working solution (1:5000 diluted in PBS) was used for staining the cells (15 min). The nuclei were then imaged using a ZOE Fluorescence Cell Imager (*λ_ex_* ~359 nm, *λ_em_* ~461 nm when DAPI is bound to DNA). Altered nuclei in the field were identified from changes in nuclear morphology/integrity (i.e., nuclear fragmentation, chromatin condensation and nuclear condensation). At least 300 nuclei were counted for each sample, including both normal and altered ones. The percentage of nucleic alteration was calculated using the following formula:

$$\%\;of\;nucleic\;alteration\;{=(Count}_{altered\;nuclei}/{Count}_{total\;nuclei})*100$$

### Secretome analysis

Transcriptome study of differentiated SGBS adipocytes treated with 100 nM S1P was previously reported [40]. We matched the differentially expressed genes (DEGs: |Fold change| ≥ 1.50 and *p* < 0.05) to the list of human secretome genes [41] to identify 36 upregulated and 21 downregulated secretome genes (Table 1).

### Statistic analysis

Data analysis was performed with GraphPad Prism 8. Data of the MTT assay were calculated from three independent experiments (n = 3) with each experiment containing six replicates. Data of the nucleic alteration (DAPI staining) were calculated from three independent experiments with each experiment containing three replicates. Results were presented as mean ± SD. Data comparison was performed using the analysis of variance (ANOVA, one-way or two-way), followed by Dunnett’s or Tukey’s post-hoc tests. Result was considered statistically significant for *p* < 0.05.

## Conclusions

In the current study, we investigated the effects of normal adipocyte-conditioned cell culture media (AD-CM) and cancer-associated adipocyte-conditioned cell culture media (CAA-CM) on S1P treatment of triple-negative breast cancer (TNBC) cells. It was observed that both AD-CM and CAA-CM mildly suppressed the anti-proliferative effect and reduced nuclear alteration/apoptosis associated with high-concentration S1P treatment. Thus, adipose tissue may be detrimental to local high-concentration S1P treatment of TNBC. Furthermore, we identified 36 upregulated and 21 downregulated secretome genes in differentiated SGBS adipocytes treated with 100 nM S1P. Most of these genes are involved in multiple biological processes, implicating that S1P may play an important role in the bidirectional communications between adipocytes and normal epithelial cells/cancer cells under physiological/pathophysiological conditions. Previous studies have shown that SphK1/S1P plays an important role in regulating stem cell functions [17, 47, 48] and obsesisty and type II diabetes [2, 49, 50]. The communication between adipocytes and different types of cells, including epithelial cells, may also be critical in regulating other physiological and pathophysiological processes in human body. Since sphingolipid metabolism pathway is highly conserved through evolution and its regulation of various biological functions is potent, further studies in S1P regulation of cell communications might provide a new clue in developing novel therapeutic agents and treating diseases such as cancer and diabetes.
